# Suffering London

**Published:** 1892-05-28

**Authors:** 


### The Hospital

An Institutional Journal of

**Science, Medicine, Nursing, and Philanthropy.**

*For Hospitals, Asylums, Infirmaries, Dispensaries, Medical Practitioners, Students, Nurses, and the Charitable Public.*

Vol. XII.—No. 296.] [May 28, 1892.

### SUFFERING LONDON.

Mr. Egmont Hake has written a book which he calls "Suffering London," and Mr. Walter Besant has acted the part of the nurse at a christening and presented the infant to the world with an appropriate introduction. It is incumbent upon us to say something about the volume; but we should be wanting in honourable candour if we did not at the outset admit a certain responsibility for the work. Our responsibility did not begin, and does not end with the mere publishing of the book. We have to confess that it is in some sort a child of The Hospital. It originated from a "forward movement," which was promoted by the Editor of this journal, acting in conjunction with the secretaries of most of the principal metropolitan charities; and a certain measure of responsibility must be taken by all those who have had any share in the attempted advance.

Having thus cleared the ground on one side it is necessary to further state that Mr. Egmont Hake is in the very fullest sense the true and proper author of "Suffering London," and that Mr. Waltert Besant is also the true and proper author of the introduction. The idea in the minds of all who have promoted the undertaking has been to make an attempt to throw an image of the voluntary hospitals of London upon a gigantic screen, so to speak, and to illuminate the picture as a whole and in each of its parts with the lime light. We are persuaded that the hospitals suffer from that familiarity which so easily breeds contempt. It is necessary, for many reasons, that all of us should put away from our minds the shadowy, imperfect, and distorted ideas we have of hospitals, and should devote to them, as they appear in the picture presented by Mr. Walter Besant and Mr. Egmont Hake, such an earnest and prolonged attention as will enable us to see them in their true light.

Newspaper writers will perhaps permit us to put a kind of suggestion and protest before them in the first instance: a suggestion that Mr. Walter Besant has earned the right to be heard on any subject which he chooses to make his own, and a protest that the hospitals, by their mere magnitude, and the nature of the work they do, are entitled to a different kind and degree of consideration from that which much smaller and less important charities can properly demand. For the sake of comparison we will put the hospitals side by side with the work of General Booth. Here let us say that, in our judgment, General Booth has done a great and much-needed work among an important and too much neglected class. But there is this fact to be pointed out, that General Booth's is only one among many large organisations which deal with the spiritual ignorance and poverty of the masses. It would be foolish and unbusiness-like for a practical people to forget the very great work that is done in this direction by various organisations supported by the Church of England, by the Congregationalists, the Baptists, the Wesleyans, and other Methodists, the Presbyterians, the Catholics, and a host of minor religious agencies. The thing to see is that all these organisations do in their measure the same kind of work as General Booth. But when we come to the great benevolent work of dealing with the sick, the injured, and the dying among the poor we find, not twenty or thirty different and wealthy organisations at work, but one organisation, and one only—the modern hospital system of medical and surgical relief.

Our contention, based on the foregoing, is that if it was right and proper for General Booth to fill the newspapers for weeks together last year, so it is right and proper for our depressed, enfeebled, and impoverished, but altogether indispensable, medical charities, to have such a share of the public attention this year as will raise them to the necessary strength and resources without which they cannot do the work they ought to do. We venture to make a most urgent appeal to the press. The book of which Mr. Besant and Mr. Hake are the sponsor and the parent is not large, it can be easily read; and if a little trouble be taken in the reading of it, the hospital system of London and the country will appear to the reader quite different from the conception he has gained from his momentary and half accidental previous glimpses at the subject.

Every newspaper writer considers it imperative that he should know a good deal about Parliament and Parliamentary affairs; he also finds it necessary to know a good deal of the Army, the Church, the Law, and, indeed, every branch of the public service. Now it is hardly too much to say that in a highly civilised empire like our own—an empire which has a task before it such as no other empire, not even the Roman, ever yet attempted—we could almost better afford to limit the operations of our legal system, or even of the Church itself, than to check and cripple the beneficent activities of our hospital system. We ask attention to Mr. Egmont Hake's proposition—that the hospitals are a fundamental and absolutely indispensable branch of the public service. Is this branch of the public service to be considered by newspaper writers and others less important and less worthy of serious attention than other branches merely because it is maintained by the spontaneous liberality of a few of our more generous citizens, and costs the State nothing? What an extraordinary method of reasoning that would be! If such an idea be correct, then the man who pays his taxes because he cannot help himself is more worthy of honour than he who voluntarily, and without the compulsion of State machinery, gives a proportion of his possessions every year for the benefit of his less fortunate fellow men.

Here, in a nutshell, is the position of affairs at the present moment. If the newspaper press of London would show the same eager activity to put the case of the hospitals before the public as it showed in putting General Booth's schemes last year, this great and indispensable branch of the public service would be lifted at once out of its paralysing poverty, and straightway endowed with the necessary vigour for doing the work that is inevitably imposed upon it. In reading through Mr. Besant's introduction and Mr. Hake's book we marked, though we have not space for, many passages which bear upon different aspects of this truly great but little understood question. Mr. Hake considers, for example, that perhaps there is no single barrier against Socialism so strong as the barrier of the hospital system. The wealthy man cannot understand why this should be so; but the working man understands it well enough. There is hardly a poor family in London which has not had some personal experience of the inside of a hospital. The father has been in with an accident, or the mother with a prolonged "weakness," or a child with a burn, or a scald, or a weak spine, or a fever. To the poor family the hospital is the ever-generous and welcome refuge when sickness pays its painful and ruinous visits to the home. Take away the hospital, and you increase the pressure of life upon the poor tenfold. So long as health remains, even poor men and women may be cheerful; and even when sickness comes, if adequate and soothing provision be made, heart and hope are not lost. But if you add to poverty a painful sickness that does not know where to turn for needed care and mitigation, you go a long way towards piling on the last burden which may drive the poor to defiance and despair.

Mr. Egmont Hake has approached the subject of "Suffering London," not from the doctor's, nor from the philanthropist's standpoint, but from the point of view of the ordinary intelligent citizen. He has done what we think every man ought to do who has a little money to spare from his annual income: he has taken the trouble to make himself thoroughly master of this great branch of the public service. That is what all pressmen at least ought to do; and what every other intelligent citizen should do who possibly can. Mr. Hake's book, whilst it does not give a hundredth part of the arguments and details which could easily be found in the subject, yet sets forth many new and fresh aspects, and presents a clear outline of the whole case. We are particularly anxious that all our readers should buy and read the volume, because, in the first place, we hope it will make enthusiastic converts of them; and also because all the profits which arise from the sale of the work will be devoted to the extension of the "forward movement" which the hospitals are now united in attempting. But, above all, are we most urgently desirous that every man who has influence with the Press shall at the present crisis do what is in him for the uplifting and permanent establishment of one of the greatest, noblest, and most absolutely indispensable "causes" of our modern times.

